# Contribution of Company Affiliation and Social Contacts to Risk Estimates of Between-Farm Transmission of Avian Influenza

**DOI:** 10.1371/journal.pone.0009888

**Published:** 2010-03-25

**Authors:** Jessica H. Leibler, Marco Carone, Ellen K. Silbergeld

**Affiliations:** 1 Department of Environmental Health Sciences, Johns Hopkins Bloomberg School of Public Health, Baltimore, Maryland, United States of America; 2 Department of Biostatistics, Johns Hopkins Bloomberg School of Public Health, Baltimore, Maryland, United States of America; Stanford University, United States of America

## Abstract

**Background:**

Models of between-farm transmission of pathogens have identified service vehicles and social groups as risk factors mediating the spread of infection. Because of high levels of economic organization in much of the poultry industry, we examined the importance of company affiliation, as distinct from social contacts, in a model of the potential spread of avian influenza among broiler poultry farms in a poultry-dense region in the United States. The contribution of company affiliation to risk of between-farm disease transmission has not been previously studied.

**Methodology/Principal Findings:**

We obtained data on the nature and frequency of business and social contacts through a national survey of broiler poultry growers in the United States. Daily rates of contact were estimated using Monte Carlo analysis. Stochastic modeling techniques were used to estimate the exposure risk posed by a single infectious farm to other farms in the region and relative risk of exposure for farms under different scenarios. The mean daily rate of vehicular contact was 0.82 vehicles/day. The magnitude of exposure risk ranged from <1% to 25% under varying parameters. Risk of between-farm transmission was largely driven by company affiliation, with farms in the same company group as the index farm facing as much as a 5-fold increase in risk compared to farms contracted with different companies. Employment of part-time workers contributed to significant increases in risk in most scenarios, notably for farms who hired day-laborers. Social visits were significantly less important in determining risk.

**Conclusions/Significance:**

Biosecurity interventions should be based on information on industry structure and company affiliation, and include part-time workers as potentially unrecognized sources of viral transmission. Modeling efforts to understand pathogen transmission in the context of industrial food animal production should consider company affiliation in addition to geospatial factors and pathogen characteristics. Restriction of social contacts among farmers may be less useful in reducing between-farm transmission.

## Introduction

The recent H1N1 pandemic draws attention to the role the organization of food animal production industries may play in the generation and transmission of novel influenza A viruses [1]–[3]. While avian influenza (AI) prevention efforts have largely focused on improving biosecurity in small-holder poultry systems in recent years, influenza risk factors associated with industrial poultry or swine production warrant increased scrutiny [4]. In nations where the poultry industry is highly industrialized and integrated by producer, such as the United States and increasingly in Asia and Latin America, the burden and transmission of infection within and among commercial flocks may serve as an important mechanism of minimizing or preventing viral adaptation and transmission to humans [5].

**Figure 1:**
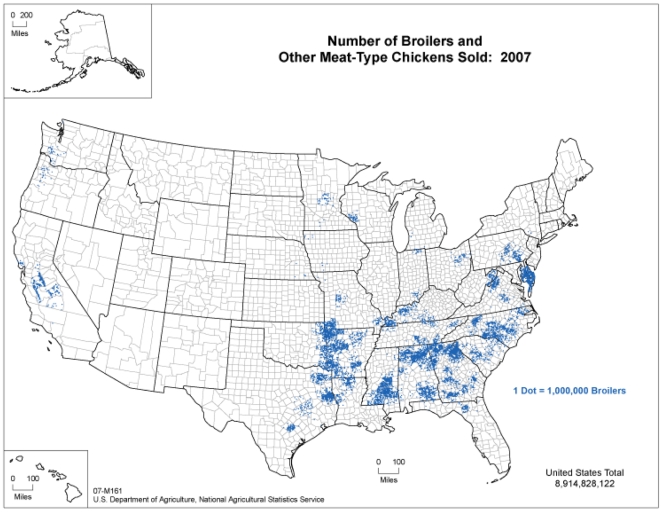
Number of broilers and other meat-type chickens sold in the United States, 2007 (Source: USDA Census of Agriculture, 2007).

More than 9 billion broiler chickens (raised for meat and slaughtered at 6–7 weeks) were produced in the US in 2007 [6]. Industrially produced poultry are raised in confined housing, provided with defined feeds rather than access to forage, and managed in order to facilitate the uniform and reliable production of meat products [7]. The industry is highly vertically integrated, with poultry production companies (known as integrators) contracting with farmers (referred to as growers) to raise the birds prior to slaughter [8]. Production is highly geographically centralized in the Southeast and Mid-Atlantic regions of the country (Figure 1).

The absence of H5N1 in the US to date does not in and of itself indicate that biosecurity measures in the US poultry industry have been effective. Low pathogenic avian influenza (LPAI) viruses of great antigenic diversity are detected frequently in commercial poultry in the US, with over 100 detections confirmed by viral isolation in commercial and small-scale flocks from 2004–2008 [9]. From 2002–2005, hemagglutinin subtypes H1-H13 and all nine neuraminidase subtypes were recovered from US poultry flocks. Prevalence of H5 and H7 LPAI (which is reported to OIE because of high pathogenic potential for poultry) in US commercial broiler and turkey flocks is estimated at .0015%, yet prevalence of non-reportable LPAI is clearly substantially higher [10].

LPAI viruses are often considered to be of lower risk because they are associated with limited mortality in poultry, but they may be harbingers of risk to humans since pathogenicity to poultry is not a precondition for human infection. Moreover, their circulation in poultry flocks provides a setting for viral evolution and human exposure.

Recent LPAI outbreaks with sustained farm-farm transmission in the US demonstrate the challenges in successfully containing AI viruses in the commercial poultry industry. A LPAI H7N2 virus infected 47 broiler flocks in Pennsylvania from 1996–1998 and seven flocks in 2001 and 2002, resulting in the culling of three million birds and losses exceeding $4 million [11], [12]. In 2002, LPAI H7N2 appeared in Virginia, spreading to 197 turkey and broiler farms and resulting in the depopulation of 4.7 million birds [13], and resurfaced in live markets and broiler facilities in the Northeast and Mid-Atlantic again in 2004. An LPAI H5N2 virus resulted in culling over 75,000 turkeys in the Shenandoah Valley in 2007; detections of LPAI H7N9 and LPAI H7N3 led to the culling of 116,000 turkeys and broiler breeders in Nebraska and Arkansas that same year. In March 2009, 20,000 broiler breeders were culled following a detection of an LPAI H7 virus in Kentucky [14].

LPAI detections in the US highlight the difficulty in preventing viral incursions in commercial poultry operations, yet likely represent a minority of the total burden of LPAI in the US. Little to no information is released to the public regarding non-H5 and non-H7 LPAI detections. Additionally, data on the poultry industry are typically not collected by state or federal agencies, and existing information is often not publically accessible. Only since 2006, when detections of low pathogenic H5 and H7 viruses became reportable to the OIE, have county-level identifiers been released to the public following a detection of LPAI in the US. At present, for non-H5 and non-H7 viruses, an infected farm is publically identified by state only; no information on specific location or company affiliation is typically released.

A growing literature supports the role of vehicular transmission and social groups as important viral conduits among farms. Two analyses of the 2002 outbreak in Virginia identified worker movement and between-farm vehicular transportation as risk factors for between–farm transmission at that time [15], [16]. In an analysis of the transmission of HPAI H7N7 from the Netherlands in 2003, Thomas et al. (2005) identified shared human resources and equipment as significant factors in international transmission of the virus [17]. Analyses of outbreaks of other zoonoses, such as foot and mouth disease and classical swine fever, have also found that vehicular transmission between farms is an important factor for pathogen movement [18], [19]. However, no previous study has considered the organization of industrial food animal production in terms of producer groups and the contribution of these groups to between-farm contact and exposure risk.

Our research aims to approximate the nature and frequency of contact patterns among poultry farms in the US through national sampling and modeling to estimate AI exposure risk in a region of high poultry density, focusing on the business dynamics specific to industrial poultry production. Quantitative modeling is useful both as a means of understanding the causes of an outbreak and to forecast risk in currently unaffected areas, and has been widely applied in the zoonotic influenza control literature [20]–[25]. The ability to use modeling to understand risk factors and control points in the US poultry industry is hampered by the lack of readily available information on rates of contact between farms. Efforts to understand viral transmission in advance of an outbreak remain critically important for AI prevention in the US.

## Methods

### Ethics statement

This research was conducted with the approval of the Johns Hopkins Bloomberg School of Public Health Institutional Review Board.

### Poultry grower survey

We developed and conducted an online survey of contract broiler growers in the US (available from the authors by request). The questions were designed in collaboration with a small group of growers in the Delmarva region who have worked with our research group on previous projects and pilot tested with this group. Our study population was a convenience sample of broiler growers who responded to an email invitation to participate in an online survey. Invitations to participate in the survey were sent to contacts and colleagues in the broiler industry, who forwarded the survey link to personal and business email lists of broiler growers. The survey was posted on the “SurveyMonkey” server, and responses were anonymous [26]. Questions were multiple choice or short answer in format. The site remained active for three months, from February 2008 to April 2008.

The survey queried growers about farm size and business cycle (number of houses, number of birds per house, total bird capacity, frequency and duration of between-flock gap periods), household size, poultry industry and service visitors to and from the farm, frequency of these visits, and biosecurity and waste management practices on the farm. Growers were asked to report how often a service vehicle affiliated with a given service visited the farm per week, per growing cycle (6–7 weeks) or per year, with the time period depending on prior knowledge of these variables. These time periods were chosen so as to maximize the usefulness of the information collected and incorporate data from farms that might be without a flock at the time of our survey.

We also asked growers if they employed workers (non-household members) to work in the poultry houses. If growers reported hiring workers, we queried the number of workers, full-time or part-time status, days per week worked during the growing season, and whether these workers worked at other poultry farms in addition to their own. Questions were also asked about frequency of social visits (family and friends), and the approximate percentage of these visitors who were also poultry growers as a way to estimate social contact between growers.

### Data analysis

For this study, we defined “integrator-linked contact” as a visit to the farm from a service vehicle or personnel affiliated with the poultry company to which the farm is contracted. “Non-integrator commercial contacts” refer to service visits from companies that are unaffiliated with a single integrator but rather service farms regardless of company affiliation. “Exposure” is defined as a visit to a susceptible farm from a vehicle that had also visited the index farm during the period of infectiousness and during the time when the virus was assumed to survive on that vehicle. To note: we focus on primary exposures from the index case in this study, rather than dynamics of viral spread once multiple farms are infected, in order to highlight upstream control points and depict the magnitude of potential exposure risk stemming from a single infectious farm.

The raw data on frequency of visits to the farm was transferred to STATA [27], and transformed into daily contact rates to permit comparisons across different vehicle sources. Daily contact rates were calculated for the following business contacts: feed delivery, flock supervisor visits (an integrator employee who is the main point of contact between the integrator and the grower), visits from other management personnel, chick delivery, live haul (removal of chickens at the end of the growing cycle for slaughter), meter readings, propane delivery, maintenance visits, cake out (removal of poultry wastes from the poultry houses) and waste haul (removal of poultry wastes from the property).

Daily contact rates were also calculated for social visits among broiler growers. Non-grower social contacts were assumed to pose less of a risk of avian influenza transmission between farms and therefore we only considered social contact among growers in our analysis.

Since a minority of respondents reported hiring part-time workers (12.5%), we estimated risk separately for farms employed part-time workers and did not include daily rates of contact with part-time workers as a risk factor for the majority of farms that did not engage in this practice. The model assumed that workers who worked only at a single poultry farm would be unlikely to expose that farm to AI from a second farm, but that workers who worked at multiple farms could transmit the virus between the farms of their employment.

The daily contact rate from part-time non-household poultry house workers was assessed using two scenarios that were intended to represent the range of likely employment practices. These scenarios were drawn from our survey data and confirmed by phone conversations and in-person interviews with Delmarva growers as appropriate for that region. The first scenario assumed uniform random mixing of part-time workers within a subset of farms who hired these workers and assumed each farm hired one worker per day. A function developed and run in the ‘R’ statistical platform [28] was used to estimate the number of distinct farms exposed by workers having visited the index farm under the assumption of random mixing during a given period. A second part-time worker scenario approximated an intermittent employment structure. Workers in this scenario worked at two distinct farms on alternating days, and each farm employed a single worker for 3 days per week.

Spearman rank correlation in STATA was used to determine correlation between continuous contact frequency data and between basic farm descriptors (number of houses, number of birds per house, and total bird capacity). This nonparametric technique was used in part due to our small sample size and evidence that our data were not normally distributed.

### Monte Carlo simulations

Daily contact rate data were transferred from STATA to an Excel spreadsheet [29]. Nonparametric bootstrapping (Monte Carlo analysis) using the Crystal Ball software package [30] was conducted for each source of contact because the data did not convincingly suggest specific parametric distributions. 10,000 simulations were conducted for daily contact rates by source, as this number of simulations was determined to be sufficient to achieve stable results. From simulation results, descriptive statistics (mean, median, standard deviation, coefficient of variation) were generated for each source of contact. Hypothesis tests and confidence intervals for estimates of contact rates, exposure risks and exposure risk differences were also constructed using nonparametric bootstrap resampling implemented in Crystal Ball.

Based on information from our grower collaborators, we considered chick delivery, live haul, feed delivery, flock supervisor visits and visits from other management personnel to circulate within the integrator group only (integrator-linked contacts). Non-integrator commercial services related to cake out, waste hauling, propane delivery and meter reading were assumed to contact farms contracted with multiple integrators and be regional, rather than company-specific, in nature. Part-time workers and grower social visits were also not limited by integrator group, under the assumption that they moved in the region unrestricted by integrator affiliation.

Sussex County, Delaware was used as the geographical framework for this analysis. Sussex County has been the top broiler producing county in the US since 1944, with production exceeding 211 million broilers in 2007 [6]. The county is situated within the Delmarva Peninsula (a region of Delaware, Maryland, and Virginia), which despite its small size (180×60 miles), produced more than 7% of total US broiler chicken production in 2007 [6]. Poultry production is densely clustered along the midline of the peninsula, and this area is ringed by four major wildlife preserves which are visited by millions of wild birds annually on the Western Atlantic flyway, posing opportunities for cross-species transmission [31]. LPAI was most recently detected in commercial poultry in Delmarva in 2004, when an LPAI H7N2 virus spread to at least three broiler farms (2 being commercial farms) before it was contained, resulting in the depopulation of over 100,000 broilers [9].

Data on the number of poultry farms in Sussex County were collected from the 2007 USDA Census of Agriculture [6] and from Google Earth maps [32]. For the Google Earth mapping, a grid was superimposed over a satellite image of the county and facilities that appeared to be poultry houses were identified by a marker, starting from a height of 38,000 feet. Three methods were used to confirm the presence and location of poultry houses: 1) a second pass through the county following major roads surrounding processing plants; 2) a third visual assessment of the grids at lower altitudes; and 3) observation and measurement of each individual farm at a lower altitude. The closer observation was intended to confirm that the object was a poultry house, identify the number of houses on the property and classify poultry houses by size (small or large). Small houses were <90 m in length and large houses were >90 m. The ruler function in Google Earth was used to measure the houses. Houses that were co-located or situated along the same driveway were considered part of a single farm.

Through Google Earth, 789 farms were identified by location and size in Sussex County, which is higher than the USDA Census estimate of 714 farms. As a result of this discrepancy, we chose an intermediate number–750 farms–as an estimate of the number of farms in the county for use in this model. Median farm size observed from our Google Earth mapping was 2 large houses, and 53% of farms had 1 or 2 large houses. 54 farms had ten or more big houses on a single property. 10% of farms had small houses only. Given a lack of consistent data on the correlation between farm size and rate of visits, however, we did not incorporate the information on farm size into our model and assumed all farms had the same rate of contact, regardless of size.

From information provided from the Delmarva Poultry Industry, Inc.[33], the regional trade organization, we assumed that there are three major integrators operating in Sussex County. We set the same number of farms to each integrator, assuming that 250 farms in the county were contracted to each integrator. There are more farms in Sussex County than other counties in the Delmarva Peninsula, but the three integrators that operate here also operate in many of the other areas in Delmarva [33].

We estimated the number of farms a single service vehicle could service in a single workday through conversations with Delmarva growers and, for non-integrator commercial operations, an assessment of the number of such firms in the region. These values were used to guide Monte Carlo simulations on these estimates, with triangular distributions fitted to these data to incorporate the minimum, maximum, and mode of our data.

Spatial factors and locational data of farms were not explicitly included in this model, which focused principally on company groups and business services. Our use of a range of farms that a given service vehicle could visit in a day implies a similar, but not exact, spatial distribution of farms within integrator groups, which does integrate spatial information a very limited way into our model. It is possible that farms associated with the same integrator are also clustered spatially, but this data was not included in our model here, which focused solely on the role of vehicular contact.

The generalizability of our contact rate data to the Delmarva region was confirmed in three ways: 1) in-person and phone conversations with Delmarva broiler growers, who reported the duration of time different service vehicles spent on the farm; 2) an analysis of truck traffic density data for Sussex County, using publically available documents from the Delaware Department of Transportation [34]; and 3) on the ground observations of truck movements in the region by the authors and our collaborators [35].

### Exposure risk estimation

Our model assumes that a single farm, contained within a single integrator group in Sussex County, becomes infected by and subsequently infectious with an AI virus. We used stochastic techniques to estimate the risk that a second farm in the region would be exposed to the virus from the infectious (index) farm during a period of infectiousness, which was calculated at a range of days (2, 5, 10, and 15 days). These time points were chosen to encompass the biological parameters of duration of infectiousness in experimental settings [36]–[41] as well as time to farmer detection and reporting of the outbreak, which may take up to 2 weeks, according to study of the 2003 HPAI outbreak in the Netherlands [42].

Data on the survival of avian influenza viruses on fomites was used to estimate the ability of a vehicle to expose secondary farm. Studies of influenza A viruses in hospital or other social settings suggest that the virus may survival on surfaces up to 2 days, with survival duration depending on surface type and temperature [43]–[45]. Research in the farm environment has observed longer survival times, up to 7 days in manure and on other farm surfaces, such as tires, feathers and plastic [46], [47]. We developed two scenarios–one assuming 2 days and one assuming 7 days of viral survival on a vehicle – to incorporate a reasonable range of survival times in our model.

Because this model is focused on exposure risk rather than infection risk, we made the simplifying assumption that the index farm had a constant level of infectiousness during its period of infectiousness, rather than assuming infectiousness followed a standard epidemic curve. This assumption added parsimony to our model and allowed for straightforward comparison of sources of risk in different scenarios.

The period-specific probability of exposure, P*_n_*(*δ*), was calculated as the risk of any given second farm in the county being contacted by a vehicle that had 1) serviced the index farm during the period of the index farm's *n* infectious days; 2) serviced the index farm before the second farm; and 3) serviced the second farm within the period during which the virus could survive on the vehicle (σ). These probabilities were calculated for farms within (*δ* = 1) and outside (*δ* = 0) the integrator group. All calculations were performed in Excel.

Information on the derivation of the equation is provided in the Supplemental Information (Figure S1) available with this article.

The probability of exposure by day *n* due to any source can be estimated by Equation 1 below, which is one minus the product of three probabilities, representing cyclical events related to integrator-linked and commercial service contacts, contacts with part-time workers, and social contact among growers.

The probability of exposure due to any source can be estimated by:
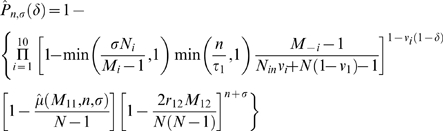



where *n* is the days of infectiousness at the index farm, *σ* is the days of viral survival on a service vehicle, *δ* is a binary variable indicating whether the potentially exposed farm is in the same integrator group as the index farm, *N_i_* is the average number of farms visited daily by vehicles in the *i* ^th^ vehicular group, *M_i_* is the number of farms serviced by a given service vehicle during the total duration of the service cycle, *τ_i_* is the average cycle length for the *i* ^th^ vehicular group, *N*_in_ is the average number of farms within integrator groups, *N* is the total number of farms in the region, *ν_i_* is a binary variable indicating whether vehicles in the *i* ^th^ vehicular group only operate within integrator groups, 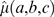
is the estimated mean number of distinct farms (excluding the index farm) exposed due to part-time workers when a total of *a* farms are participating in worker exchanges, and with periods of farm and vehicle infectiousness of *b* and *c* days, respectively, *r_12_* is the average daily number of social grower-visitors at a given visitor-receiving farm, and M_12_ is the average number of visitor-receiving farms on any given day.

## Results

Seventeen broiler growers completed our online survey (n = 17). Characteristics of survey respondents are presented in Table 1. Respondents represented basic demographics of the broiler industry in terms of size of farms and poultry houses and regional distribution. The median number of chickens per house was 23,500, with a median of 4 houses per farm. Seventy three percent (73%) of respondents were from Southeast states, with 20% from the mid-Atlantic region and the remaining 7% from the Midwest and West.

**Table 1:** Select characteristics of survey respondents*.

Variable	Values
Median number of broilers per house	23,500 (range: 15,500–100,000)
Median number of broiler houses on the farm	4 (range: 2–12)
Median total bird capacity	100,800 (range: 33,700–400,000)
Median household size	2 adults
% of respondents reporting:	
Hiring non-household workers on the farm	10 farms
Hiring part-time workers+	2 farms
Caking out the poultry houses themselves or with help of other growers	14 farms
Doing some or all of poultry house maintenance themselves	14 farms
Full-time or part-time off-farm employment for self or spouse	10 farms
Employment in the poultry industry for 10+ years	10 farms

*n = 17 respondents.

+ n = 16 for this question.

No significant correlations among our daily contact rate calculations were observed by visit type, lending support to our assumption that contact rate variables are independent. As a measure of farm size, total bird capacity at one time on the farm was positively correlated with flock supervisor visits (p = 0.045), as well as with a composite variable representing all non-integrator commercial visits (p = 0.013) and marginally with feed delivery visits (p = 0.069).

Daily contact rates, total and by source, are presented in Table 2. The mean daily contact rate from all sources was 0.82 vehicular contacts per day. Integrator sources accounted for approximately 80.5% of total contact, with 15.5% of contacts connected to non-integrator commercial visits and part-time workers. 4.0% of total contacts were social visits among growers. According to these results, a broiler farm in our sample is visited by a contact linked to the integrator approximately at least once every 1.5 days and by a contact linked to non-integrator commercial services every 8 days. Growers in our survey reported a social contact with another grower an average of once per month.

**Table 2:** Daily vehicular contact rates at a broiler farm, by source.

Source of contact	Mean daily rate of contact (5^th^, 95^th^ percentile)*	Approximate mean frequency of visits (range; max-min)+	Coefficient of variation*
**Integrator-linked contacts**			
Feed delivery	0.48 (0.20, 1.00)	2 days (1–5 days)	0.61
Flock supervisor	0.12 (0.02, 0.16)	9 days (6–45 days)	0.35
Chick delivery	0.03 (0.02, 0.04)	35 days (23–45 days)	0.35
Live haul	0.03 (0.02, 0.04)	35 days (23–45 days)	0.35
Management personnel other than flock supervisors	0.01 (0.00, 0.04)	142 days (22 days–no visits)	1.94
**Total from integrator group**	**0.66 (0.31, 1.20)** ∧ **(80.5% of total)**	**1.5 days (2x daily–3 days)**	**0.45**
**Non-integrator commercial visits and part-time workers**			
Propane delivery	0.04 (0.02, 0.07)	26 days (15–45 days)	0.39
Meter reading	0.04 (0.02, 0.09)	26 days (11–45 days)	0.70
Maintenance/repair	0.01 (0.00, 0.07)	83 days (23 days- no visits)	1.60
Waste hauling	0.01 (0.00, 0.02)	167 days (65 days–no visits)	0.93
Cake out	0.01 (0.00, 0.01)	180 days (120–360 days)	0.24
Part-time workers	0.03 (0, 0.10)	37 days (1 day–no visits)	1.35
**Total, non-integrator commercial visits and part-time workers**	**0.13 (0.08, 0.27)** ∧ **(15.5% of total)**	**8 days (5–19 days)**	0.40
**Grower social contacts**	**0.03 (0.00, 0.10) (4.0% of total)**	**30 days (10 days–no visits)**	**0.77**
**Total daily contact, by all sources**	**0.82 (0.45, 1.36)** ∧	**1.2 days (2x daily–2 days)**	**0.37**

*Values derived from survey data from national survey of poultry growers and Monte Carlo simulations. Confidence intervals generated through nonparametric bootstrapping resampling in Crystal Ball.

+ Based on daily contact rate.

∧ Sums calculated from data presented above differ slightly from totals presented due to rounding.

Feed delivery accounted for the highest rate of contact, occurring at each farm once every 2 days during the 6–7 week growing cycle. The flock supervisor visited each farm on average every 9 days during the growing cycle. Other management personnel were reported to visit the farm less frequently, on average 2–3 times during the year. Contact from chick delivery and live haul vehicles occurred typically 1–2 times during the growing cycle, with farms with more chickens reporting dual delivery and pickup cycles.

Propane deliveries and meter readings occurred approximately monthly (mean every 26 days). Mean frequency for visits from outside maintenance or repair services was low–approximately 4–5 times per year–with 82% of farmers reporting doing some or all repairs themselves (Table 1). Daily contact from cake out and waste hauling service contributed minimally to the total daily contact rate, with each service occurring 2–3 times annually and 82% of farmers reporting caking out their poultry houses on their own and not hiring outside companies for this service.

### Contribution to variance

Rate of visits from non-flock supervisor management personnel, maintenance/repair workers and part-time workers had the highest coefficient of variation estimates, suggesting greater variability in the data for these two variables than for other sources of contact (Table 2). Rates of cake out service visits had the lowest contribution to variance, followed by flock supervisor, chick delivery and live haul services.

### Exposure risk estimates

**Figure 2:**
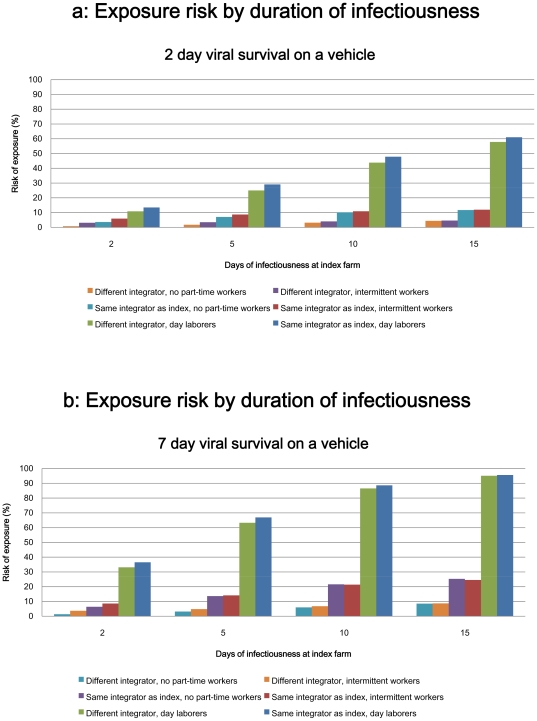
Exposure risk by duration of infectiousness and viral survival on vehicle.

The model estimated the risk of exposure for farms within the same integrator group as the index farm and for farms outside the same integrator group (that is, contracted to other integrators) but within Sussex County. Point estimates of risk and 95% confidence intervals are presented in Table 3 and in bar graph form in Figure 2a and 2b.

**Table 3:** Estimated exposure risks for second farm, given one infectious farm in region.

	Point estimates for risk of exposure (%) for a second farm given a single infectious farm in the region (95% confidence interval)			
	2 days of viral survival on service vehicle		7 days of viral survival on service vehicle	
Number of infectious days at index farm	Shared integrator affiliation with as index farm	Different integrator	Shared integrator affiliation with as index farm	Different integrator
**2 days**	3.6% (2.4–4.9)	0.75% (0.3 - 1.0)	6.4% (4.2–8.6)	1.4% (0.93–2.1)
**5 days**	7.1 (3.6–11.5)	1.8 (1.5–2.0)	13.6 (8.1–19.4)	3.2 (2.1–5.0)
**10 days**	10.1 (6.8–14.5)	3.2 (2.6–4.3)	21.6 (15.6–27.8)	6.0 (3.9–9.7)
**15 days**	11.8 (8.3–16.2)	4.4 (3.6–6.0)	25.3 (18.7–32.3)	8.5 (5.5–14.0)

*These probabilities include integrator-linked service visits, visits from non-integrator commercial services, part-time workers and social contacts among growers. Confidence intervals generated through nonparametric bootstrapping resampling in Crystal Ball.

Assuming a 2-day period of viral survival on a vehicle, a second farm affiliated with the same integrator group as the index farm would likely face an exposure risk of more than 3% with a 2 day period of infectiousness at the index farm [mean  = 3.6 (95% CI: 2.4, 4.9)] (Table 3). This risk may exceed 11% at 15 days of infectiousness [mean = 11.8; (8.3, 16.2)]. Farms outside the integrator group of the index farm faced minimal risk at 2 days of infectiousness of the index farm (<1%) and upwards of 4% risk of exposure by 15 days of infectiousness [mean = 4.4 (3.6, 6.0)].

In the scenario assuming 2 days of viral survival on a vehicle, farms within the same integrator group as the index farm faced nearly 5-fold increase in risk compared to farms associated with different integrator groups at 2 days of infectiousness [RR  = 4.9 (3.1, 6.8)]. At 15 days of infectiousness of the index farm, farms within the same integrator group had a greater than 2-fold increase in risk of exposure [RR  = 2.7 (1.9, 3.8)] (Table 4).

**Table 4:** Relative risk of exposure, by integrator group.

	Relative risk of exposure for farms within the same integrator group as the index farm (95% CI)*		
Number of infectious days at index farm	2 days of viral survival on a service vehicle	7 days of viral survival on a service vehicle	
**2 days**	4.9 (3.1, 6.8)		4.9 (3.0, 7.4)
**5 days**	4.1 (2.2, 6.8)		4.5 (2.5, 7.1)
**10 days**	3.2 (2.1, 4.7)		3.8 (2.4, 5.6)
**15 days**	2.7 (1.9, 3.8)		3.1 (2.1, 4.5)

*Reference population is a farm that is not affiliated with the integrator group of the index farm at the same model parameters (viral survival and duration of infectiousness). Confidence intervals obtained using nonparametric bootstrapping resampling in Crystal Ball.

If 7 days of viral survival on a vehicle is assumed (which might occur as a result of manure on tires, for example [46], [47]), farms within the same integrator group as the index farm may face an exposure risk greater than 6% at 2 days of infectiousness of the index farm [mean  = 6.4 (4.2, 8.6)]. This risk may exceed 25% at 15 days of infectiousness [mean  = 25.3(18.7, 32.2)]. Farms affiliated with different integrator groups had minimal risk at 2 days of infectiousness (<2%), with the exposure risk at 15 days of infectiousness approximately 8% [mean  = 8.5 (5.5–14.0%)].

In the 7 day viral survival scenario, farms within the integrator group had an approximately 5-fold increase in risk compared to farms affiliated with different integrators [RR = 4.9 (3.0, 7.4)]. These farms faced a greater than 3-fold increase in risk at 15-days of infectiousness (RR = 3.1 (2.1, 4.5).

Being within the same integrator group as the index farm was therefore associated with statistically significant increases in exposure risk for farms under all model parameters (p<0.05) (Table 4).

Point estimates of risk varied significantly with the model parameters of duration of infectiousness at the index farm and duration of viral survival on a vehicle parameters (p<0.05). In all scenarios, however, the effect of being within the same integrator group as the index farm contributed to a greater relative increase in risk than did changes to the model parameters of duration of infectiousness at the index farm duration of viral survival on a vehicle.

Hiring day laborers was associated with statistically significant increases in exposure risk across all model parameters (p<0.05) (Table 5). Hiring intermittent workers also contributed to statistically significant increases in exposure risk for farms that engaged in this practice across most scenarios, but the increase in risk associated with this practice was notably less than that associated with hiring day laborers. In both part-time employment scenarios, relative risk of exposure was significantly higher for farms that had a different integrator affiliation than the index farm compared to farms within the same integrator group as the index farm (p<0.05). Risk estimates in the part-time worker scenarios assume that the index farm also engages in the given employment practice.

**Table 5:** Relative risk of exposure for farms that hire part-time workers (95% confidence interval)*.

	2 day viral survival on a service vehicle			
	Same integrator group as index farm		Different integrator group	
Days of infectiousness at index farm	Day laborer scenario	Intermittent worker scenario	Day laborer scenario	Intermittent worker scenario
2 days	3.8 (3.0, 5.2)	1.7 (1.5, 2.0)	14.7 (11.4, 17.0)	4.2 (3.4, 4.7)
5 days	4.5 (2.8, 6.8)	1.3 (1.1, 1.4)	14.5 (11.1, 17.0)	2.0 (1.7, 2.2)
10 days	4.9 (3.5, 6.7)	1.1 (1.1, 1.1)	13.9 (10.4, 16.6)	1.3 (1.2, 1.3)
15 days	5.4 (3.9, 7.1)	1.0 (1.0, 1.0)	13.4 (9.8, 16.2)	1.0 (1.0, 1.1)

	7 day viral survival on a service vehicle			
	Same integrator group as index farm		Different integrator group	
Days of infectiousness at index farm	Day laborer scenario	Intermittent worker scenario	Day laborer scenario	Intermittent worker scenario
2 days	5.9 (4.4, 8.2)	1.4 (1.3, 1.5)	25.8 (15.9, 35.2)	2.8 (2.1, 3.5)
5 days	5.3 (3.6, 8.0)	1.1 (1.1, 1.2)	21.2 (12.6, 29.4)	1.6 (1.3, 1.8)
10 days	4.2 (3.2, 4.6)	1.0 (1.0, 1.0)	15.5 (9.0, 22.0)	1.1 (1.1, 1.2)
15 days	3.9 (3.0, 5.1)	1.0 (1.0, 1.0)	12.1 (6.9, 17.4)	1.0 (1.0, 1.0)

*Reference group is farms who do not hire part-time workers with the same model parameters (viral survival on a service vehicle, days of infectiousness, and integrator affiliation).

A farm outside the integrator group of the index farm that hired day-laborers faced a greater than 13-fold increase in risk compared to farms that did not in the 2 day viral survival scenario (Table 5). In the 7 day viral survival scenario, the farms hiring day laborers had a 12- to 25-fold increase in risk compared to farms that did not hire day laborers. Farms within the same integrator group as the index farm hiring day laborers had at a greater than 3-fold increase in risk compared to farms in the integrator group that did not hire day laborers.

Hiring intermittent part-time workers contributed to marginal to small increases in risk for farms within the same integrator group as the index farm, with relative risk ranging from 1.0 to 1.7 under varying parameters. This practice contributed to higher relative risk of exposure for farms that were not in the same integrator group as the index farm, with a greater than 4-fold increase in risk observed in one scenario [RR:4.2 (3.4, 4.7]. By 15 days of infectiousness at the index farm, the practice of hiring intermittent workers did not contribute significantly to elevated risk compared to farms that did not hire these workers regardless of integrator affiliation due to visits by other service personnel during this longer time period.

The percent contribution to overall risk by each source of vehicular contact was also estimated (Table 6). With 2 days of index farm infectiousness and 2 days of viral survival on a vehicle, sources of contact from within the integrator group accounted for nearly 85% of total exposure risk for farms within the group. Non-integrator commercial visits contributed approximately 4% of total exposure risk, and part-time workers contributed 11% of total risk. Social visits among growers accounted for less than 1% of total risk. With a longer period of infectiousness of the index farm and a vehicle, the relative contribution of the integrator group is reduced, with the non-integrator sources accounting for more than 17% of exposure risk. The contribution of part-time workers and social visits to total risk decreased minimally with this change in assumptions.

**Table 6:** Percent contribution to total exposure risk, by source of contact.

	Contribution to total exposure risk (%)	
	2 days of farm infectiousness, 2 days of vehicle infectiousness	15 days of farm infectiousness, 7 days of vehicle infectiousness
**Integrator-linked contacts**		
Feed delivery	73.7	32.7
Flock supervisor	6.83	19.1
Chick delivery	2.3	10.9
Other management personnel	0.0	0.0
Live haul	1.9	9.1
**Total**	**84.7**	**71.8**
**Non-integrator commercial services**		
Meter reading	1.1	5.0
Propane delivery	2.2	10.3
Maintenance	0.0	0.0
Cake out	0.2	0.8
Waste haul	0.2	1.1
**Total, non-integrator commercial services**	**3.7**	**17.2**
**Part-time workers**	**10.9**	**10.4**
**Grower social contacts**	**0.8**	**0.7**
**Total, all sources**	**100.0**	**100.0**

At 2 days of farm infectiousness and viral survival on a vehicle, feed delivery visits are the predominant source of risk of exposure, accounting for nearly 74% of total risk for farms within the same integrator group as the index farm, followed by the part-time workers and flock supervisors, which contributed approximately 11% and 7% respectively to total risk. The other sources of contact contributed minimally to total exposure risk during a short duration of infectiousness and viral survival. At 15 days of farm infectiousness and 7 days of vehicle infectiousness, additional sources contribute more significantly to overall risk and the role of feed delivery is reduced. Feed delivery and flock supervisor visits still play a prominent role in risk, contributing more than 50% of risk together, but other sources–including chick delivery, part-time workers, propane delivery and live haul–contribute together more than 40% of total exposure risk for these farms.

## Discussion

Our analysis indicates that company affiliation is a major driver of farm-based exposure risk to an infection like avian influenza in region with high-density food animal production. Farms within the same integrator group as the index farm may face as much as a 5-fold increase in exposure risk compared to farms affiliated with a different integrator.

This “integrator effect” is stronger for short duration of infectiousness and for short periods of viral survival on vehicles, due to the high farm contact rate with integrator-linked services, but statistically significant at all model iterations (p<0.05). Sources of farm contact associated with the integrator group contributed from 72–85% of total exposure risk, depending on scenario.

Hiring part-time poultry house workers was also observed to be a significant risk factor for exposure, particularly the practice of employing day laborers (who were assumed to move randomly among farms that engaged in this practice). Intermittent workers (who worked at set multiple farms on a regular schedule) also contributed to increases in exposure risk for farms that hire these workers, but the relative risks associated with this practice were significantly less than those associated with hiring day laborers.

Social contacts among growers do not appear to drive exposure risk in our model, accounting for 1% or less of total contribution to risk.

Our results indicate that a single infectious farm within the context of a dense, broiler producing region can result in quantifiable AI exposure risk to other farms as a result of vehicular business contacts. In a real-world setting, where it may take up to 2 weeks to detect an LPAI outbreak in an industrial flock and a virus can persist for long periods of time in manure [42], [46], [47], farms associated with the same integrator as the index farm may face a 25% risk of exposure to a vehicle that had serviced the index farm during its period of infectiousness. Farms affiliated with different integrators may have an exposure risk upwards of 8%. While the point estimates should not be taken as interpretations of precise risk, they suggest that the risk posed by vehicular business contacts is a potentially significant source of viral transmission in a poultry-dense region.

These results suggest that attention to the economic structure of the poultry industry, specifically integrator-level groups and business practices, may be critically important in estimating the risk of outbreak in areas dominated by industrial-scale animal production. Given the geographic consolidation of the industrial food animal industry–especially in the US, where the industry is highly concentrated in a few regions–models that estimate risk in these regions must specifically consider factors unique to business connections and practices in this industry. Geospatial models that focus solely on distance among farms as the primary risk factor for disease transmission may not capture the full dynamics of disease spread in settings where production is dominated by a vertically integrated structure and industrial food animal production methods.

Correlation analyses found that certain contact rates were significantly and positively associated with farm size as measured by total bird capacity. Correlations were observed between total bird capacity and flock supervisor visits, feed delivery and non-integrator commercial visits. As these variables were important predictors of risk in our model, these correlations suggest that total bird capacity may be a useful proxy for risk. However, correlations were not observed using other measures of farm size (physical size of poultry house, number of growing cycles per year, and total number of houses on the property). A similar study conducted in Georgia did not find a correlation between farm size and rate of visits; more research is required in this area to better inform modeling efforts [48].

The coefficient of variation estimates suggest that the rates of visits from management personnel other than the flock supervisor, maintenance/repair services and part-time workers have the highest relative variability of all sources of contact considered in the model. These results suggest that the greatest need for more information lies in these areas, and that future studies considering contact patterns among poultry farms should pay particular attention to these sources of contact. Low variability in cake out visits, flock supervisor visits, chick delivery, live haul and propane suggest more consistency in these values among farms.

Our results are supported by findings from other studies that have reported that human movement and equipment sharing among poultry farms is an important factor in the spread of AI viruses [15], [16], [17], [49]. Our results are also largely in concordance with Vieira et al. (2009), who conducted a similar survey of poultry growers in Georgia, US [48].

As any stochastic model consists of a simplified representation of reality, we made a number of key assumptions in our analysis. For example, we did not include environmental sources of transmission (wild animal movement, or wind or water transport, for example) or viral emissions in our model. A growing literature supports the notion that confined animal facilities pose biosecurity risks, and that pathogen movement can occur through high throughput ventilations systems, water emissions, insect and rodent movement, and waste management practices, among other mechanisms [50]–[54]. While difficult to quantify, these emissions are undoubtedly important in transmission and our model likely underestimates true exposure risk by excluding them.

We did not weigh contacts by level of risk, making the assumption that all business-related or grower social contacts to the farm pose an equal level of risk of pathogen transmission. Further studies aimed at weighing risk of various contacts would be valuable in this regard. Our study did not incorporate possible differences in biosecurity practices by farmers, integrators and businesses, which may be important mediating factors in a true outbreak situation. Our model also does not include information on contacts within the poultry industry involving other products (layer chickens, turkeys, ducks, etc) or contacts between these industries. Future projects could consider area where there is overlap by poultry industries, such as North Carolina, to better estimate risk in these regions.

A central limitation of this study is the small sample size of our survey from which our estimates were generated for modeling purposes. Our use of Monte Carlo analysis was geared towards providing variance measures on these estimates. It is feasible that our sample population is biased, and that growers with access to the internet or who participate on email listserves may be fundamentally different than growers who do not. The demographics of our respondents coincide with overall industry demographics and observations from the Vieira et al. study however, suggesting this concern is less germane [6], [48].

As previously noted, this study focuses on risk of exposure, rather than infection. Infection involves multiple factors, including viral adaptation to the host species [55], dose [39], [56], route of exposure [57] and viral survival in different environments [58], [59] among other factors. There is inherent variability and uncertainty in estimating risk of infection, particularly from novel viruses. Modeling exposure risk can help target interventions, but predictions of infection transmission require knowledge of viral and host parameters. We made the choice in this model to focus on exposure risk rather than infection risk due to our prevailing interest in exploring the role of company affiliation in risk and using our survey data to estimate this risk. Limiting the current model to exposure allowed us to highlight this area of interest while avoiding uncertainties involved in modeling the full course of an infectious disease in a farm population. It would be expected that risk of actual infection would be less than the exposure risk estimates we calculate, under the assumption that not every exposure results in infection.

Our study was intended to be a caricature of a poultry dense region and inform general assessments of exposure risk and high-risk practices, rather than a definitive depiction of exposure risk. Our intention was to provide an initial exercise into infectious disease modeling for AI transmission in the context of US broiler farms, and in doing so, we hope to encourage additional data collection and research in this area of relevance to industrial scale food animal production, which is increasingly practiced around the world. Our analysis could be performed with data from other areas or industries to identify practices of high risk and target interventions, and could be used as the basis for models that consider the full course of an outbreak.

### Policy implications

Business contacts are cyclical and reliable sources of potential pathogen transmission, and as such, can be controlled through guidelines or regulations. The integrator group is the locus of risk of exposure from vehicular traffic, and therefore, heightened attention should focus on improving and maintaining biosecure practices for integrator vehicles. Regular disinfection of these vehicles or reducing the frequency of services may be more straightforward and efficient than targeting other sources of potential transmission, such as confinement house emissions or wild animal movement.

In addition to large trucks or service vehicles, this study suggests that passenger cars–such as those driven by flock supervisors and workers–may play a significant role in exposure risk, and it is important to include them in biosecurity and biocontainment plans.

The practice of hiring part-time workers in the poultry industry should also be considered in analyses of biosecurity and biocontainment. Integrators and growers engaging in these practices should take care to disinfect personal vehicles and provide protective equipment to growers for use by workers–particularly work clothing that can be laundered at an appropriate facility, rather than at home (the latter of which is commonplace in the industry) [60].

Our analysis of social contact among growers suggests that exposure risk from social contact is low. Therefore, the social quarantine of growers at infected farms may not have a significant impact in reducing transmission of the virus. However, the rate of social contacts between growers in our study was notably less than that observed by Vieira et al. in Georgia, and further research is required to elucidate social contact patterns among grower populations in different regions [48].

Interventions focused on halting business related vehicular contact within the integrator group of the infected farm and quarantining the group, without regard to distance between farms, would improve biosecurity and biocontainment efforts in areas with high density animal production. Control strategies targeted specifically at banning waste hauling services within a radius around an infected farm, as was mandated by the US state of Virginia in 2007 following an LPAI detection, may not significantly reduce the risk of outbreak because of the infrequency of this activity. Attention to the frequency of visits by different services when designing biosecurity and biocontainment strategies would likely improve the efficacy of these efforts.

The effect of shared integrator group was a stronger driver or risk in our model, emphasizing the need for data on industry associations in addition to other parameters in future modeling exercises in industrialized regions. Modeling efforts focused on between-farm pathogen transmission in the context of industrial food animal production should explicitly include data on integrator groups, rather than just spatial factors. As the industrial model of food animal production spreads throughout the world and expands to other food animal industries (notably swine), attention to the specific practices in this industry is imperative to the successful control of zoonotic disease.

Company affiliation may serve as a disease transmission network for poultry facilities, particularly those located in high-density production regions in the US. Knowledge of basic industry dynamics can help identify risk profiles for these areas. Standardized collection and greater availability of this data in the US is essential to preventing AI outbreaks in commercial poultry flocks and subsequent human transmission; keeping this data confidential undermines animal and human health goals. Existing data on LPAI outbreaks should be centralized and publicized–including information on specific integrators involved in outbreaks–to aid in prevention efforts.

## Supporting Information

Figure S1 Model specifications and derivation of probability equations.
(0.08 MB PDF)
