# Supplementary material for: Contribution of Company Affiliation and Social Contacts to Risk Estimates of Between-Farm Transmission of Avian Influenza
Source: PLoS One. 2010 Mar 25;5(3):e9888. doi: 10.1371/journal.pone.0009888 (PMC2845626; doi:10.1371/journal.pone.0009888)
Supplement: Figure S1 — Model specifications and derivation of probability equations. (0.08 MB PDF) [file pone.0009888.s001.pdf]

Contribution of company networks and social contacts to risk estimates of between-farm transmission of avian influenza. Leibler, J.H., Carone, M., and Silbergeld, E.K.

SUPPLEMENTAL INFORMATION

Model specification:

The possible causes of exposure for a farm in the region are $C_1, C_2, \ldots, C_{12}$. Causes $C_1, C_2, \ldots, C_{10}$ (feed delivery, flock supervisor, chick delivery, live haul, management personnel, propane delivery, meter reading, maintenance/repair, waste hauling and cake out, respectively) are cyclical events, in that we assume that they occur at regular intervals throughout the growing cycle or the year concordant with our data. Of these, $C_1, C_2, \ldots, C_5$ are assumed to operate within an integrator network only and $C_6, C_7, \ldots, C_{10}$ operate without regard to integrator affiliation. We made the assumption that these services occurred within closed groupings of farms that were serviced by the same vehicle in an ordered manner, to simulate a service cycle by a given service vehicle. As a result of this assumption, vehicles do not move randomly among farms in the region but rather visit a set number of farms in a particular order that maintains the frequency of visits depicted by our data. These vehicle groupings either existed within the integrator network (for causes $C_1, C_2, \ldots, C_5$) or across the region, encompassing farms in different integrator networks (for causes $C_6, C_7, \ldots, C_{10}$).

As described in the main text, we estimated the number of farms that a given service vehicle (by source) could service per day using Monte Carlo analysis based on input provided by grower collaborators. The results of these simulations, in conjunction with our contact rates by source, were used to calculate the number of vehicles operating within the integrator network or region pertaining to each source.

The assumption of a highly structured set of constraints on commercial visits and service vehicles is reasonable based on the highly integrated business model of the poultry industry, which is to ensure a continuous delivery of a set number of market-ready poultry products to each processing plant.

Causes C_{11} and C_{12} (part-time workers and grower social visitors) were treated differently than the other causes in the model. Contact from these causes were not assumed to be cyclical but rather took on random mixing patterns or established probabilities of contact, as specified in the sections below. Based on survey data, we assumed that only a small percentage of farms employed part-time workers - who were assumed to work at multiple farms - and created a subset of farms in the model within which part-time workers would mix. As described below, we developed two scenarios to model the movement of part-time workers: one simulating a day-laborer scenario and one simulating an intermittent worker scenario.

Social visits between growers were assumed to occur at frequencies described by our data. We assumed that a social visit consisted of a meeting between two growers from different farms at one of their homes, but our approach could be adapted to other patterns as well.

To note, our model considered exposure risk from vehicular contact with a commercial service vehicle, part-time worker, or social visitor. The vehicle itself serves as a fomite in our model.

Denote by A the infectious farm and by B another farm of interest. Denote by Δ a binary variable indicating whether A and B are in the same integrator group. Denote by $E_{i,n,\sigma}$ the event that B is exposed due to C_i with periods of farm and vehicle infectiousness of n and σ days, respectively, and by $F_{i,k}$ the event that B is exposed due to C_i on day k. For $i = 1, 2, \ldots, 11$, denote by S_i the event that A and B

are in the same C_i-group, i.e., that A and B are serviced by the same vehicle with respect to cause C_i, and by $V_{i,n}$ a binary variable indicating whether A has been serviced relative to cause C_i no later than day n. Denote by $T_{12,k}(C)$ a binary variable indicating whether farm C takes part in social visits on day k.

We make the following assumptions:

1. For any i and j distinct, $E_{i,n,\sigma}$ and $E_{j,n,\sigma}$ are independent, i.e., exposure from different causes is independent.
2. For any $i = 1, 2, \ldots, 10$, Δ and $E_{i,n,\sigma}$ are independent given S_i and $V_{i,n}$, i.e., if membership in specific vehicle's service area as well as index farm service status are known, common integrator group membership and exposure are independent.
3. For any $i = 1, 2, \ldots, 10$, Δ and $V_{i,n}$ are independent given S_i, i.e., if membership in specific vehicle's service area is known, index farm service status and common integrator group membership are independent.
4. For any $i = 11, 12$, Δ and $E_{i,n,\sigma}$ are independent, i.e., integrator group membership and exposure are independent.
5. The variables $F_{12,1}, F_{12,2}, \ldots$ are independent random variables, i.e., day-specific exposures are independent of each other.
6. For each cause, a service vehicle has equal probability of visiting any of the visitable farms.
   - In the case of cyclical causes $C_1, C_2, \ldots, C_{10}$, visitable farms at a given instant by a given vehicle are the farms in the service area of this vehicle not having been visited yet.
   - For cause C_{11}, visitable farms for a given part-time worker include all farms hiring part-time workers and not having been visited by this farmer in the same day.
   - For cause C_{12}, visitable farms are all those partaking in social visits.

Our goal is to compute the all-cause probability of primary exposure for another farm of interest, given an assumed period of infectiousness at a single index farm and an assumed duration of viral survival on a service vehicle or car (vehicle infectiousness) that services the index farm..

The all-cause probability of exposure associated to n days of farm infectiousness and σ days of vehicle infectiousness, which we denote by $\mathrm{P}_{n,\sigma}(\delta)$, may be computed as

$$\begin{aligned}
\mathrm{P}_{n,\sigma}(\delta) &= \mathrm{P}\Big(\cup_{i=1}^{12} E_{i,n,\sigma} \mid \Delta = \delta \Big) \;=\; 1 - \mathrm{P}\Big(\cap_{i=1}^{12} \bar{E}_{i,n,\sigma} \mid \Delta = \delta \Big) \\
&= 1 - \prod_{i=1}^{12} \Big[1 - \mathrm{P}\left(E_{i,n,\sigma} \mid \Delta = \delta \right) \Big].
\end{aligned}$$

However, for $i = 1, 2, \ldots, 10$, we may write that

$$\begin{aligned}
\mathrm{P}\left(E_{i,n,\sigma} \mid \Delta = \delta \right) &= \mathrm{P}\left(E_{i,n,\sigma} \cap V_{i,n} \cap S_i \mid \Delta = \delta \right) \\
&= \mathrm{P}\left(E_{i,n,\sigma} \mid V_{i,n} \cap S_i \right) \mathrm{P}\left(V_{i,n} \mid S_i \right) \mathrm{P}\left(S_i \mid \Delta = \delta \right) \;,
\end{aligned}$$

while $\mathrm{P}\left(E_{11,n,\sigma} \mid \Delta = \delta \right) = \mathrm{P}\left(E_{11,n,\sigma} \cap S_{11} \right) = \mathrm{P}\left(E_{11,n,\sigma} \mid S_{11} \right) \mathrm{P}\left(S_{11} \right)$ and

$$\begin{aligned}
\mathrm{P}\left(E_{12,n,\sigma} \mid \Delta = \delta \right) &= \mathrm{P}\left(E_{12,n,\sigma} \right) \;=\; 1 - \prod_{k=1}^{n+\sigma} \Big[1 - \mathrm{P}\left(F_{12,k} \right) \Big] \\
&= 1 - \prod_{k=1}^{n+\sigma} \Big[1 - \mathrm{P}\left(F_{12,k} \cap T_{12,k}(B) \cap T_{12,k}(A) \right) \Big] \\
&= 1 - \prod_{k=1}^{n+\sigma} \Big[1 - \mathrm{P}\left(F_{12,k} \mid T_{12,k}(B) \cap T_{12,k}(A) \right) \mathrm{P}\left(T_{12,k}(B) \mid T_{12,k}(A) \right) \mathrm{P}\left(T_{12,k}(A) \right) \Big] \;.
\end{aligned}$$

As such, we obtain our final expression for $\mathrm{P}_{n,\sigma}(\delta)$ as

$$1 \quad - \quad \left\{ \prod_{i=1}^{10} \Big[1 - \mathrm{P}\left(E_{i,n,\sigma} \mid V_{i,n} \cap S_i\right) \mathrm{P}\left(V_{i,n} \mid S_i\right) \mathrm{P}\left(S_i \mid \Delta = \delta\right) \Big] \times \Big[1 - \mathrm{P}\left(E_{11,n,\sigma} \mid S_{11}\right) \mathrm{P}\left(S_{11}\right) \Big] \right.$$
$$\left. \times \prod_{k=1}^{n+\sigma} \Big[1 - \mathrm{P}\left(F_{12,k} \mid T_{12,k}(B) \cap T_{12,k}(A)\right) \mathrm{P}\left(T_{12,k}(B) \mid T_{12,k}(A)\right) \mathrm{P}\left(T_{12,k}(A)\right) \Big] \right\} \ .$$

Parameter estimation:

Denote by N_{in} the average number of farms within an integrator group, and by N the total number of farms in the region considered (i.e., union of all integrator groups).

Consider cause C_i for $i = 1, 2, \ldots, 10$. Denote by N_i the average number of farms visited daily by a single vehicle in the ith vehicular group, by M_i the average number of farms serviced by any particular vehicle across the days of its service cycle, and by τ_i the length of the service cycle associated to the ith vehicular group. We may estimate the probability $\mathrm{P}\left(E_{i,n,\sigma} \mid V_{i,n} \cap S_i\right)$ of exposure given that the index farm has been visited within n days relative to cause C_i and that the farm of interest is serviced by the same C_i-vehicle group as the index farm, for general n, by

$$\hat{\mathrm{P}}\left(E_{i,n,\sigma} \mid V_{i,n} \cap S_i\right) \ = \ \min\left(\frac{\sigma N_i}{M_i - 1},\ 1 \right) \ .$$

The probability $\mathrm{P}\left(V_{i,n} \mid S_i\right)$ that the index farm will be visited in n days relative to cause C_i given that the index farm and the farm of interest are serviced by the same C_i-vehicle may be estimated by

$$\hat{P}\left(V_{i,n} \mid S_i\right) \ = \ \min\left(\frac{n}{\tau_i},\ 1 \right) \ .$$

The probability of being serviced by the same C_i-vehicle given whether the potentially exposed farm is in the same integrator group as the index farm can be estimated by

$$\hat{\mathrm{P}}(S_i \mid \Delta = \delta) = \begin{cases} (M_i - 1)/(N_{\text{in}} - 1) & : \ \delta = 1 \\ 0 & : \ \delta = 0 \end{cases} ,$$

if service vehicles operate within integrator groups alone. Otherwise, $\hat{\mathrm{P}}(S_i \mid \Delta = \delta) = (M_i - 1)/(N - 1)$ is the appropriate estimator.

Next, we consider cause C_{11}. The conditional exposure probability $\mathrm{P}\left(E_{11,n,\sigma} \mid S_{11}\right)$ may be estimated by

$$\hat{\mathrm{P}}\left(E_{11,n,\sigma} \mid S_{11}\right) \ = \ \frac{\mu(M_{11}, n, \sigma)}{M_{11} - 1} \ ,$$

where $\mu(a, b, c) = \mathrm{E}[Z_a(b, c)]$ and $Z_a(b, c)$ is a random variable indicating the number of distinct farms (excluding the index farm) exposed due to part-time workers when a total of a farms are participating in worker exchanges, and with periods of farm and vehicle infectiousness of b and c days, respectively. Here, M_{11} is the average number of farms hiring part-time workers daily.

We consider the following two scenarios:

(A) Among farms hiring part-time workers, the latter are randomly reassigned on a daily basis, with assignment probability equal for all available farm-worker combinations. Assignments on any two given days are independent and all farms have exactly one part-time worker per day.

(B) Among farms hiring part-time workers, exactly half are visited by a single worker at any given time. Each worker visits exactly two farms on alternating days, with three full days spent at a given farm in each week.

Under scenario (A), the distribution (and hence mean) of $Z_a(b,c)$ may be obtained by the following Monte Carlo algorithm:

1. Create array of a rows and $b+c$ columns;
2. For $i \in \{1,2,\ldots,b+c\}$, set column i of the array to be a random reordering of digits $(1,2,\ldots,a)$;
3. For $j \in \{1,2,\ldots,b\}$, identify the digit stored in row 1 and column j of the array. List the row numbers at which the latter appears between columns $j+1$ and $j+\sigma$. Set $Z_a(b,c)$ to be the number of distinct row numbers obtained upon aggregating the above list for each j;
4. Repeat steps 1-3 many times and use observations to empirically estimate the distribution (and in particular, the mean) of $Z_a(b,c)$.

Under scenario (B), calculations simplify greatly. Since the worker involved with the index farm works at exactly one additional farm (on alternating days), only one farm will be exposed. Thus, in this scenario, we may write $\hat{\mu}(a,b,c) = \mu(a,b,c) \equiv 1$ for $a,b,c > 1$.

The probability $\mathrm{P}(S_{11})$ may be estimated by $\hat{\mathrm{P}}(S_{11}) = (M_{11}-1)/(N-1)$.

Finally, we consider cause C_{12}. The conditional exposure probability $\mathrm{P}(F_{12,k} \mid T_{12,k}(B) \cap T_{12,k}(A))$ can be shown to be estimated by

$$\hat{\mathrm{P}}(F_{12,k} \mid T_{12,k}(B) \cap T_{12,k}(A)) \;=\; \frac{2r_{12}}{(r_{12}+1)[(r_{12}+1)M_{12}-1]} \,,$$

where r_{12} and M_{12} are the average number of visitors per day at a given visitor-receiving farm and the average number of visitor-receiving farms on a given day, respectively. The probabilities $\mathrm{P}(T_{12,k}(B) \mid T_{12,k}(A))$ and $\mathrm{P}(T_{12,k}(A))$ may themselves be estimated by

$$\hat{\mathrm{P}}(T_{12,k}(B) \mid T_{12,k}(A)) \;=\; \frac{(r_{12}+1)M_{12}-1}{N-1} \quad \text{and} \quad \hat{\mathrm{P}}(T_{12,k}(A)) \;=\; \frac{(r_{12}+1)M_{12}}{N} \,.$$

Substituting the above estimators in our main question yields the estimator

$$\hat{\mathrm{P}}_{n,\sigma}(\delta) \;=\; 1 - \left\{ \prod_{i=1}^{10} \left[1 - \min\left(\frac{\sigma N_i}{M_i - 1},\, 1 \right) \min\left(\frac{n}{\tau_i},\, 1 \right) \frac{M_i - 1}{N_{\mathrm{in}}\nu_i + N(1-\nu_i) - 1} \right]^{1-\nu_i(1-\delta)} \times \left[1 - \frac{\hat{\mu}(M_{11}, n, \sigma)}{N-1} \right] \times \left[1 - \frac{2r_{12}M_{12}}{N(N-1)} \right]^{n+\sigma} \right\},$$

where ν_i is a binary variable indicating whether service vehicles operate only within integrator groups for the ith vehicular group.
